# Selenoprotein N and SEPN1-Related Myopathies: Mechanisms, Models, and Therapeutic Perspectives

**DOI:** 10.3390/biom16010125

**Published:** 2026-01-12

**Authors:** Martina Lanza, Ester Zito, Giorgia Dinoi, Antonio Vittorio Buono, Annamaria De Luca, Paola Imbrici, Antonella Liantonio, Elena Conte

**Affiliations:** 1 Department of Experimental Medicine, University of Salento, 73100 Lecce, Italy; martinaa.lanzaa@gmail.com; 2 Istituto di Ricerche Farmacologiche Mario Negri IRCCS, 20156 Milan, Italy; ester.zito@uniurb.it; 3 Department of Biomolecular Sciences, University of Urbino Carlo Bo, 61029 Urbino, Italy; 4 Department of Pharmacy-Drug Sciences, University of Bari “Aldo Moro”, 70125 Bari, Italy; giorgia.dinoi@uniba.it (G.D.); a.buono24@phd.uniba.it (A.V.B.); annamaria.deluca@uniba.it (A.D.L.); paola.imbrici@uniba.it (P.I.)

**Keywords:** myopathy, Selenoprotein N, skeletal muscle, ER stress

## Abstract

Selenoprotein N (SelN or SELENON) is a selenium-containing protein of the endoplasmic/sarcoplasmic reticulum (ER/SR), encoded by the *SEPN1* gene. In skeletal muscle, SelN is particularly important for regulating SR calcium homeostasis. It acts as a calcium sensor, modulating the activity of the sarcoplasmic reticulum calcium pump (SERCA) through a redox-dependent mechanism. Loss-of-function mutations in the *SEPN1* gene give rise to a spectrum of skeletal muscle disorders collectively referred to as SEPN1-related myopathies (SEPN1-RM). Histopathologically, SEPN1-RM is characterized by the presence of minicores, which are localized regions within muscle fibers exhibiting mitochondrial depletion (i.e., cores) and sarcomeric disarray. As no effective therapy is currently available for SEPN1-RM, understanding SelN biology through loss-of-function models remains essential for elucidating disease mechanisms and identifying potential therapeutic targets. This review examines the current knowledge on SelN function and the pathological mechanisms underlying *SEPN1* loss-of-function, with a particular focus on the connection between calcium handling, oxidative/ER stress, and muscle dysfunction. It also highlights emerging strategies aimed at restoring SelN activity or mitigating downstream defects, outlining potential therapeutic avenues for SEPN1-RM.

## 1. Introduction

Selenoproteins are a class of proteins that contain the essential trace element selenium in the amino acid selenocysteine (Sec). Selenium is an essential micronutrient with a wide range of biological functions, including the regulation of gene expression, immune function, and antioxidant defense mechanisms [1]. It serves as a key component of various selenoproteins involved in cellular signaling, redox balance, and other critical physiological functions [2]. Selenium typically exists in nature in inorganic forms, such as selenium acid, selenite, or elemental selenium, which can be converted into organic seleno compounds by living organisms. These different forms of selenium have varying degrees of bioavailability and can be metabolized and utilized by the body in different ways [3]. SelN was among the first selenoproteins identified through computational methods [4] and is involved in maintaining cellular homeostasis by protecting against oxidative damage [5]. Particularly, SelN contributes to several cellular activities, including redox regulation, protein folding, and calcium signaling modulation. SelN is abundantly expressed in skeletal muscle and is causative of specific neuromuscular conditions [4,6,7,8]. Since its discovery in 1999, mutations in the gene encoding SelN have been associated with four autosomal recessive, early-onset muscle diseases: rigid spine muscular dystrophy (RSMD1), multi-minicore disease (MmD), congenital fiber type disproportion (CFTD), and desmin-related myopathy with Mallory body-like inclusions (MB-DRMs). These conditions are now collectively classified as Selenoprotein N1 (SEPN1)-related myopathy (SEPN1-RM). Despite advances in understanding SelN’s physiological role in muscle, the pathogenic molecular mechanisms driving SEPN1-RM are still not fully understood.

In this review, we provide an overview of SEPN1-RM with a particular focus on SelN expression patterns and function in skeletal muscle. We also recall how its loss-of-function gives rise to SEPN1-RM involving ER stress, calcium dyshomeostasis and metabolism, thus offering insights for the development of therapeutic strategies.

## 2. Clinical and Pathophysiological Features of SEPN1-RM

**Figure 1:**
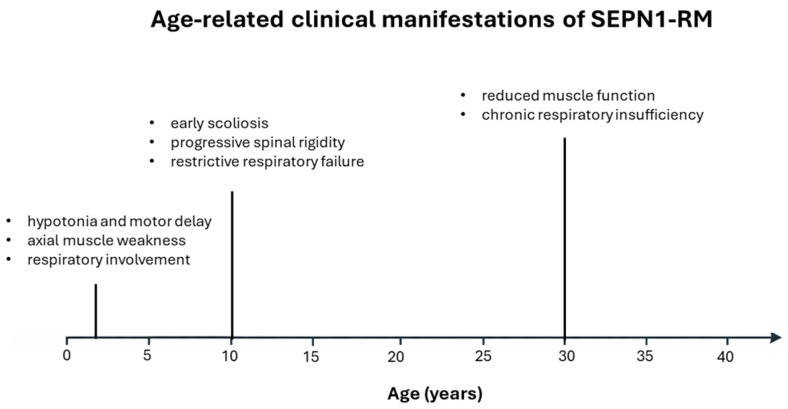
Timeline illustrates the typical age-related onset and progression of major clinical features in SEPN1-RM, from early childhood to adulthood.

SEPN1-RM is an ultrarare disorder with an unknown prevalence. It is estimated to account for 11.65% of congenital muscular dystrophy cases [9] and 16% of congenital myopathies [10]. Only ~130–150 cases have been reported in large international cohorts, and there appears to be no significant gender bias, with proportions of males and females being roughly equal (approximately 50.8% female and 49.2% male in one international cohort of 132 patients) [11]. The disorder presents a highly recognizable and relatively homogeneous clinical phenotype. Symptoms typically appear within the first two years of life in nearly 80% of cases, manifesting as hypotonia, weakness of the neck and trunk, and axial muscle involvement accompanied by respiratory impairment. Before adulthood, disease progression commonly included scoliosis and spinal rigidity, with respiratory failure becoming significant and often necessitating nighttime non-invasive ventilation. In the third decade of life, patients are usually still ambulatory but are characterized by chronic muscle weakness and persistent respiratory insufficiency [11,12] (Figure 1).

Patients typically display a characteristic facial appearance, a long and slender neck, a flat and retracted thorax, severe axial weakness, and progressive spinal rigidity. Axial muscles, particularly the diaphragm, are especially susceptible to oxidative stress, a direct consequence of SelN depletion [13]. Although axial weakness and rigidity are usually present from early childhood, they are underrecognized because limb strength and ambulation remain relatively preserved for many years [14,15]. As a result, clinical suspicion is often delayed until the end of the first decade, when scoliosis and restrictive respiratory failure become evident.

Disease severity is further influenced by genetic and metabolic factors, with biallelic null *SEPN1* mutations associated with more severe phenotypes. Increased adiposity from childhood has also been linked to rapid disease progression, early loss of ambulation, and extra-muscular involvement, possibly reflecting defects in mitochondrial bioenergetics and lipid metabolism [16,17]. Furthermore, studies show that SelN depletion promotes insulin resistance and saturated fatty acid-induced toxicity, leading to chronic SR stress and worsening muscle weakness [16]. Consequently, routine use of hypercaloric or high-fat diets may be harmful and should be avoided in the absence of clear evidence of malnutrition.

Despite its clinically uniform presentation, SEPN1-RM displays considerable histopathologic heterogeneity. Muscle biopsies most frequently show features consistent with congenital myopathy, including focal depletion on Nicotinamide Adenine Dinucleotide Tetrazolium Reductase (NADH-TR) and Succinate Dehydrogenase (SDH) staining, indicative of mitochondrial dysfunction, as well as minicore lesions characterized by localized sarcomeric disorganization and mitochondrial depletion. Histological analysis of skeletal muscles commonly shows nonspecific myopathic alterations, such as a predominance of type 1 fibers, marked fiber size variability, and nuclear atrophy [18], although moderate dystrophic changes and protein aggregates may also be present. Histopathologic severity does not correlate with clinical progression; rather, it is largely influenced by the muscle sampled, with severely dystrophic axial muscles often contrasting with only mildly affected limb muscles in the same patient. Moreover, biopsies obtained at younger ages are associated with less specific features, suggesting that muscle biopsy should be reserved for diagnostically challenging cases and may be more informative when performed at school age [11].

A recent longitudinal study indicates that SEPN1-RM is more severe and progressive than previously assumed [11]. While motor function is often considered stable, some patients lose ambulation, and both skeletal muscle and respiratory function begin to decline from the third decade, even in mild cases, with reports of early mortality and reduced lifespan. Disease outcomes are strongly influenced by respiratory and orthopedic management: early noninvasive ventilation may stabilize respiratory decline [19], and scoliosis surgery can improve long-term respiratory function, despite a transient post-operative reduction in forced vital capacity.

This clinical picture is exemplified by a recent case report of a 15-year-old boy with SEPN1-RM, who experienced axial hypotonia and mild scoliosis, multi-minicore myopathy and progressive sleep-disorder breathing evolving into severe obstructive sleep apnea with hypoventilation. Despite conventional noninvasive ventilation, his nocturnal oxygen desaturation persisted until a personalized volume-targeted pressure support mode was implemented, which effectively normalized oxygenation and reduced respiratory events [20].

Taken together, these findings highlight the need for a multidisciplinary approach to patient management, involving coordinated medical, respiratory, rehabilitative, and nutritional interventions aimed at preserving motor and respiratory function and improving long-term outcomes.

## 3. Synthesis, Structure and Localization of SelN

In 1998, a rare form of congenital muscular dystrophy characterized by early-onset spinal rigidity, known as RSMD1, was linked to chromosome 1p35–p3 [21,22]. A few months later, the gene encoding for SelN was identified through in silico analysis and found to be associated with multiple cases of RSMD1 [4].

**Figure 2:**
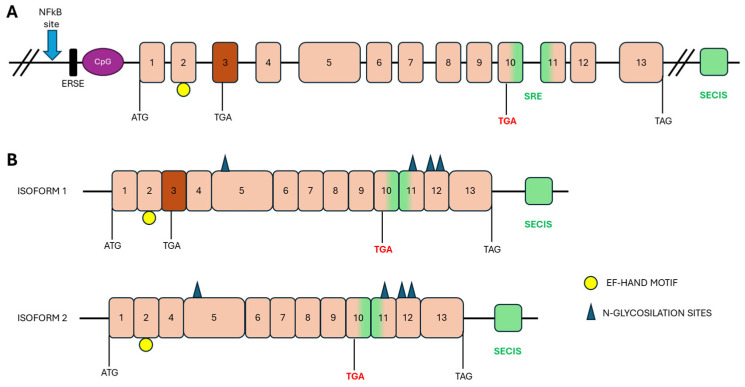
Schematic representation of *SEPN1* gene and its isoforms. (**A**) Genomic organization of the human *SEPN1* gene. Upstream of the translation start site are the non-transcribed regions of the gene: the NF-kB binding site, the ERSE region and the CpG islands. The positions of the start (ATG), stop (TAG) and Sec (TGA in red) codons are indicated below the gene. Exons are shown in pink boxes; exon 3, alternatively spliced, is shown in dark orange. The SECIS in the 3′UTR and the SRE within the coding region are shown in green. (**B**) Two isoforms of the transcript differ in the splicing of exon 3. The positions of the selenocysteine incorporation site (TGA in red) and the translation start (ATG) and termination (TAG) codons are indicated. Predicted motifs: Putative N-glycosylation sites are indicated by blue triangles and the EF-hand motif by a yellow circle.

In humans, *SEPN1* gene is located on chromosome 1p35–p36, and its orthologs have been identified in all vertebrates and some invertebrates, exhibiting strong conservation of protein sequences, especially in the amino acids surrounding the Sec residue [23]. The gene’s coding sequence comprises 13 exons, with the TGA-Sec codon residing in exon 10 (Figure 2A). Notably, exon 3 contains an Alu sequence, which is unique to primates, and undergoes alternative splicing to produce two transcript isoforms: a short and a long isoform (Figure 2B). Both isoforms are expressed in skeletal muscle, brain, lung, and placenta [24]. While the long isoform contains two potential in-frame Sec incorporation sites, the short isoform encodes only one Sec residue and is the only one expressed. Indeed, the long isoform does not produce a functional protein due to an in-frame TGA codon in exon 3, which is recognized as a stop codon [24,25,26].

Sec (one-letter code U) is the 21st amino acid in the genetic code. In eukaryotes, Sec residue is inserted by Sec-tRNA, which is encoded by the TGA codon, into the polypeptide chain by a complex machinery that includes cis- and trans-factors. The cis-elements include the Sec insertion sequence (SECIS) in the 3′UTR of eukaryotic selenoprotein mRNAs [27,28]. Some selenoproteins, such as SelN, possess a hairpin structure called the Sec codon redefinition element (SRE), downstream of the TGA codon, which facilitates Sec insertion. These elements, along with Sec-tRNA 1q and ribosomes, interact with key proteins, such as the translation factor SECIS-binding protein 2 (SBP2) and the Sec elongation factor (EFSec), to enable Sec incorporation and ensure translation [29,30]. The recognition of the Sec codon as a premature stop leads to the destabilization of the *SEPN1* transcript. The Sec insertion mechanism appears to have a dual role: it promotes the insertion of a Sec residue into the polypeptide chain and protects the selenoproteins from degradation. While it remains unclear which of these mechanisms is activated when the Sec residue insertion mechanism is impaired, one study has identified SBP2 as critical in determining the sensitivity of selenoprotein mRNAs to nonsense decay [31]. The Sec residue is more reactive in nucleophilic reactions than cysteine, suggesting that selenoproteins play a significant role in protecting cells against oxidative stress.

**Figure 3:**
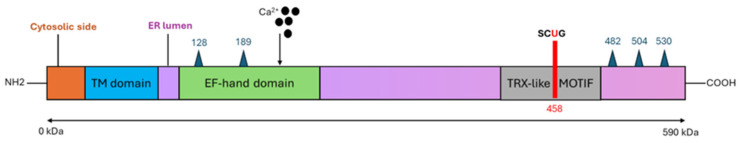
Schematic representation of SelN protein. SelN is a type II transmembrane glycoprotein of the ER. The figure illustrates the glycosylation sites (blue triangles), the NH2-terminal part protruding into the cytosolic side (orange part), the transmembrane region of the ER (blue box), and both the calcium-binding EF-hand domain (in green) and the thioredoxin-like motif (in gray) protruding into the ER lumen, which carries incorporated Sec in position 458 (the SGUC redox motif).

The protein encoded by *SEPN1* gene consists of 590 amino acids, with a molecular mass of 68 kDa (Figure 3). The peptide sequence contains four predicted glycosylation sites (positions 155, 448, 470 and 496), a calcium-binding domain (EF-hand) and proline residues organized into eight PXXP motifs, consistent with protein/protein interactions through SH3 protein domains [32,33]. The promoter region of SelN contains multiple NF-κB binding sites and a putative endoplasmic reticulum stress response (ERSE), suggesting that SelN may be regulated in response to inflammatory conditions, oxidative stress and endoplasmic reticulum (ER) stress [34]. SelN is a single-spanning integral membrane protein with a short N-terminus located in the cytosol; most of the protein, including the U of the active site, is situated in the lumen of the ER. The Sec residue is at amino acid 458 within a redox SCUG motif, reminiscent of the motif found in thioredoxin reductases (GCUG). This motif constitutes the catalytic site of the protein, conferring oxidoreductase activity [8,35,36].

## 4. The Multifaceted Role of SelN in Cellular Function

**Figure 4:**
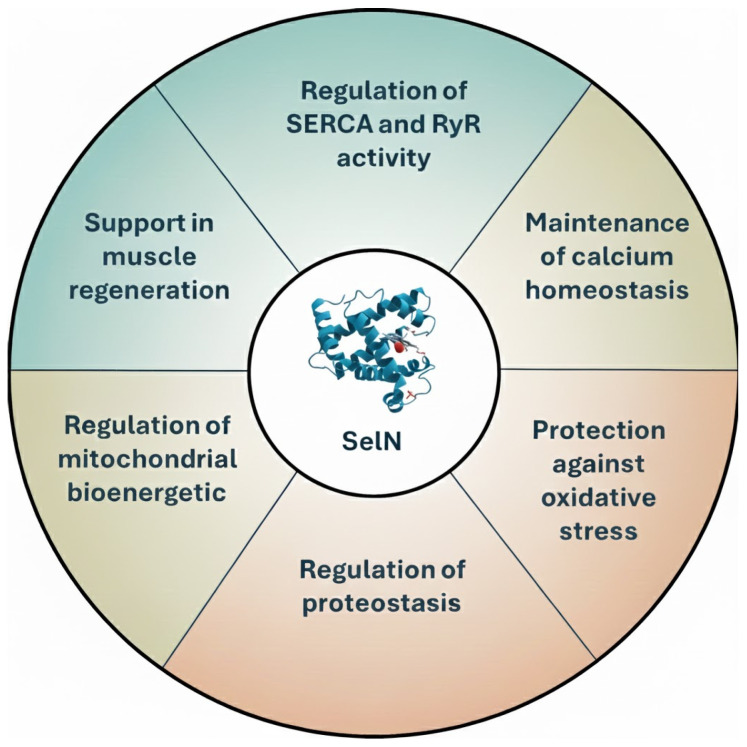
Schematic summary of the main functions of SelN.

SelN is in the ER membrane, where it acts as both a Ca^2+^ sensor and a redox regulator. Its distinct N-terminal domain enables it to bind to and monitor Ca^2+^ levels within the ER lumen, playing a pivotal role in maintaining ER Ca^2+^ homeostasis and overall cellular function. Disruptions in SelN expression or function can lead to severe skeletal muscle disorders, including stiff spine muscular dystrophy and myopathy, highlighting its critical role in maintaining the structural and functional integrity of muscle tissue [37]. Furthermore, SelN has been shown to be involved in regulating the cellular stress response, especially in the context of ER stress. Emerging evidence highlights that SelN is essential for modulating redox signaling pathways within the ER, thereby mitigating the harmful effects of ROS and helping to maintain protein folding homeostasis [6,33]. Thus, SelN is a multifunctional selenoprotein crucial for maintaining calcium homeostasis, redox balance, and proteostasis within the ER (Figure 4). Elucidating the mechanisms through which SelN exerts its protective functions will be key to developing therapeutic strategies for a range of skeletal muscle, ER and metabolic disorders.

### 4.1. Pathways Regulated by SelN and Related Dysfunctions in SEPN1-RM

#### 4.1.1. ER Stress

SelN is a key reticular-resident selenoprotein that contributes significantly to maintaining ER equilibrium, particularly by alleviating ER stress, a cellular disturbance caused by the buildup of improperly folded or unfolded proteins within the ER lumen. Its role in regulating intracellular calcium levels and redox conditions is fundamental for efficient protein folding and overall cell health [24]. When SelN function is compromised, cells, especially those in muscle and neural tissues, become more vulnerable to ER stress, which has been linked to disorders such as SEPN1-RM [37].

A central pathway through which SelN influences ER stress involves its regulation of the unfolded protein response (UPR). This adaptive mechanism restores ER function by activating three stress sensors: IRE1 (inositol-requiring enzyme-1), ATF6 (activating transcription factor 6), and PERK (protein kinase RNA-like ER kinase) [38]. These sensors initiate a cascade of signaling events that reshape gene expression, protein synthesis, and post-translational modifications, ultimately aiming to reduce the accumulation of misfolded proteins and re-establish proteostasis [38,39]. However, chronic unresolved ER stress can lead to cellular dysfunction and apoptosis due to maladaptive activations of the stress response. All three pathways activate pro-apoptotic signals via the transcription factor CHOP (GADD153), which drives ER stress-induced apoptosis [40]. CHOP upregulates genes such as ERO1α, which promotes protein folding by forming disulfide bonds into nascent proteins but also generates H_2_O_2_, a dangerous oxidant. Additionally, CHOP recruits the phosphatase PP1 (GADD34) to dephosphorylate eIF2α, thereby resuming protein synthesis under stress [41,42,43].

In SelN-deficient muscle, as seen in SEPN1-RM such as rigid spine muscular dystrophy (RSMD1) and multi-minicore disease, prolonged ER stress is a consistent pathological feature, with muscle biopsies showing multi-minicores, mitochondrial depletion, and sarcomeric disorganization [44]. The absence of SelN exacerbates oxidative stress, prolongs UPR activation, and promotes apoptosis [8]. Notably, inhibition of ERO1α can protect against ER stress-induced toxicity in SEPN1-deficient myotubes, indicating a potential therapeutic strategy for the related myopathy.

Given its central role in ER stress regulation and its clear involvement in the muscle degeneration seen in SEPN1-RM, SelN represents a promising therapeutic target for diseases characterized by chronic ER stress and oxidative imbalance. Further research is warranted to clarify the molecular mechanisms by which SelN exerts its protective effects and to develop targeted therapies that modulate its function in stress-related diseases.

#### 4.1.2. Calcium Homeostasis

In healthy muscle, proper regulation of Ca^2+^ within the SR is critical for contraction, and disturbances in this regulation can trigger SR stress, leading to muscle dysfunction and atrophy [45]. The SR communicates with mitochondria through mitochondria-associated membranes, known as MAMs, critical for different cellular pathophysiological processes, including Ca^2+^ transport, lipid metabolism, mitochondrial dynamics, cellular stress responses (including ER stress and oxidative stress), ATP production and regulation of cell death pathways [7,46]. SelN is enriched in the MAMs region of skeletal muscle, where it regulates Ca^2+^ balance and mitochondrial bioenergetics [7,47,48]. Loss of SelN triggers ER/SR stress, alters MAMs architecture, disrupts SR Ca^2+^ levels, and impairs mitochondrial membrane potential, leading to reduced Ca^2+^ uptake and ATP synthesis [7,49]. These alterations may partly account for the metabolic and systemic phenotypes observed in patients with SEPN1-RM [7].

Mechanistically, SelN binds Ca^2+^ via its EF-hand domain, activating SERCA-mediated Ca^2+^ reuptake and protecting it from oxidative inactivation. In SelN-deficient muscle, oxidative stress from hyperactive ERO1α further inhibits SERCA, prolonging muscle relaxation time, altering the distance between ER and mitochondria, and impairing contractility [6,49,50]. The hypothesis that Ca^2+^ microdomains regulate the distance between the SR and mitochondria suggests a role for this interaction in controlling mitochondrial metabolism and ATP production [51]. SelN deficiency reduces Ca^2+^ uptake into the SR and prolongs muscle fiber relaxation time after electrical stimulation, indicating impaired Ca^2+^ refilling of the SR. SelN regulates ER Ca^2+^ handling and redox balance by activating SERCA to replenish the ER/SR Ca^2+^ pool in skeletal muscle [6,50,52].

Clinically, the disruption of Ca^2+^ homeostasis is reflected in the hallmark features of SEPN1-RM as well as in histopathology showing type 1 fiber predominance and mitochondrial depletion. Progressive scoliosis and respiratory failure are common, likely linked to chronic Ca^2+^-handling defects in diaphragm muscle [53]. Satellite cell depletion, observed in SelN-deficient mouse models, further impairs regenerative capacity, triggering functional decline [54].

Thus, the role of SelN at the intersection of redox control and Ca^2+^ signaling is central to both the molecular pathogenesis and the clinical phenotype of SEPN1-RM.

#### 4.1.3. Metabolism

Skeletal muscle plays a key role in glucose metabolism, serving as the primary site for insulin-dependent glucose uptake (75–90%). However, lipid accumulation in muscle can be lipotoxic, inducing SR stress and impairing insulin signaling. These findings suggest that SR stress and maladaptive responses can contribute to muscle atrophy, weakness and altered metabolic function [55,56].

In SEPN1-RM patients, metabolic alterations are often observed alongside muscle weakness, including abnormal Body Mass Index (BMI), altered fat distribution, and insulin resistance in severely underweight individuals. Body weight correlates with disease severity: underweight patients often maintain ambulation into adulthood, while overweight patients lose it earlier, possibly due to exacerbated oxidative stress and Ca^2+^ dysregulation.

In SelN-deficient cells, ER stress might trigger insulin resistance [49]. Furthermore, SelN deficiency in animal models recapitulates extra-muscular metabolic phenotypes observed in SEPN1-RM patients, with lipid accumulation, impaired glucose handling, and reduced mitochondrial respiratory chain activity [7,57]. This suggests that beyond its structural role in muscle, SelN is a metabolic regulator whose dysfunction contributes to both systemic and muscle-specific features of SEPN1-RM.

## 5. Mutations in SEPN1 and Clinical Spectrum of SEPN1-RM

**Figure 5:**
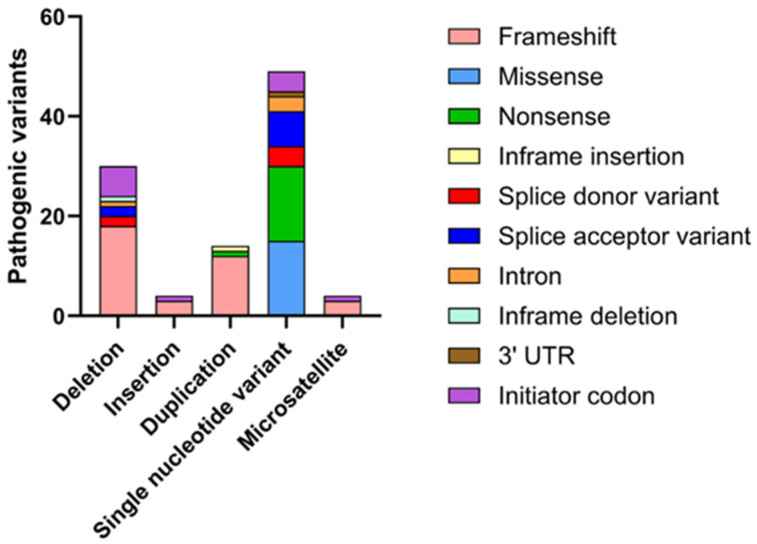
Graphical representation of the main mutations affecting the *SEPN1* gene on ClinVar. Based on a search on ClinVar, approximately 93 mutations with a pathogenic phenotype have been identified, the majority of which are associated with RSMD1. These mutations have been categorized according to the type of variation (deletion, insertion, etc.), and for each, the molecular consequence (frameshift, nonsense, etc.) has been specified.

Mutations in the *SEPN1* gene are associated with a spectrum of congenital myopathies, collectively known as SEPN1-RM, and are distributed across the entire *SEPN1* gene. Due to the rarity of the disease, the exact incidence of SEPN1-RM remains uncertain. The majority of these mutations consist of nonsense mutations, micro-deletions, and insertions that result in frameshifts, as well as splice-site mutations that cause abnormal pre-mRNA splicing (Figure 5).

These mutations follow an autosomal recessive inheritance pattern and are expected to result in a loss of function. Furthermore, several single-nucleotide variations leading to missense mutations are concentrated around the predicted catalytic site, which is identified by the presence of a Sec residue, or affect conserved residues across vertebrate evolution from fish to humans [58].

A clinical study involving 132 patients worldwide demonstrated that over half of the identified mutations were homozygous, while about one-third were compound heterozygous. Most mutations were located in exons 1, 6, 7 and 11, with exon 1 being the most frequently affected and associated with a more severe phenotype. Furthermore, a study involving Chinese patients with RSMD1 identified several novel mutations, primarily affecting the transmembrane and thioredoxin domains of SelN. These included frameshift mutations, such as c.7_8insGGGCC (p.Arg5Glyfs*63) and c.233delC (p.Ser78Serfs*21), leading to truncated, nonfunctional proteins. Missense mutations, such as c.1384T>C (p.Sec462Arg) and c.1397G>A (p.Arg466Gln), were also identified, affecting critical regions like the SCUG motif, which is essential for the protein’s catalytic activity [59]. Another notable mutation, c.943G>A, has been associated with congenital fiber-type disproportion, a condition characterized by the relative hypotrophy of type 1 muscle fibers. This mutation underscores the diverse phenotypic manifestations that can arise from different *SEPN1* mutations [60]. Furthermore, mutations affecting the selenocysteine insertion sequence (SECIS) element in the 3′ untranslated region (UTR) of *SEPN1* mRNA have been reported. For example, a homozygous point mutation g.17195T>C disrupts the SECIS element, impairing the incorporation of Sec into SelN. This disruption significantly reduces protein levels, triggering the development of SEPN1-RM [61]. The pathogenicity of these mutations is primarily attributed to their detrimental effects on the structure and function of SelN. Loss-of-function mutations often result in truncated proteins lacking essential domains required for maintaining redox homeostasis and calcium regulation within muscle cells. Missense mutations can alter the protein’s conformation, affecting its stability and interaction with other cellular components. Additionally, mutations in regulatory regions like the SECIS element can hinder proper protein synthesis, leading to insufficient levels of functional SelN. A comprehensive knowledge of the spectrum of *SEPN1* mutations and their specific impacts on protein function is crucial for elucidating the molecular mechanisms underlying associated myopathies. This knowledge not only supports accurate genetic diagnosis but also paves the way for the development of targeted therapeutic strategies aimed at mitigating the effects of these mutations.

Research into the connection between *SEPN1* mutations and muscle pathology remains ongoing, with studies indicating that disrupted interactions between the ER and mitochondria, along with impaired muscle regeneration, are key mechanisms underlying the disease [7].

## 6. SEPN1-RM Animal and Cellular Models

### 6.1. Animal Models

SEPN1-RM refers to a group of muscle disorders that, while clinically similar, vary in severity. Currently, no specific pharmacological treatment exists, and the underlying pathophysiological mechanisms remain poorly understood. In this regard, the development of animal models that accurately mimic the human biochemical defect is a critical step forward. SelN is widely expressed across tissues, with peak expression during early development, particularly in the myotome during embryogenesis. It is believed to play a role in myoblast proliferation and muscle organization. Notably, SelN expression decreases during in vitro myoblast differentiation and myotube formation [14].

Several animal models have been employed to study embryonic development, using antisense morpholino oligonucleotides and, more recently, CRISPR/Cas9 technologies [58,62,63] (Table 1). Zebrafish from Deniziak’s group exhibits somite disorganization and severe alterations in overall muscle architecture, while the Jurynec model shows defects in slow fiber development and reduced expression of myogenic genes in adaxial cells [58,62]. When these SelN-deficient adaxial cells are transplanted into wild-type embryos, they form abnormal slow fibers, indicating a cell-autonomous effect. However, the specific contributions of SelN deficiency to these phenotypes, as well as potential off-target effects of morpholinos, remain unclear. To address this, Barraza-Flores’ group generated a stable germline SelN knockout zebrafish model, which exhibits changes in embryonic muscle function and swimming activity in larvae. Single-cell RNA sequencing from a zebrafish embryonic atlas revealed co-expression of SelN with genes involved in the glutathione pathway and redox homeostasis, suggesting a functional link among them [63].

To explore the in vivo role of SelN and the consequences of its deficiency, Rederstorff’s group developed a SelN knock-out mouse model in 2011, generating the first mammalian model for SEPN1-RM. In contrast to zebrafish, these mice showed no abnormalities in somitogenesis or expression of myogenic factors [64]. Adult SelN-deficient mice do not develop overt muscular disorders and appear similar to wild-type animals. However, they show mild muscle alterations and increased susceptibility to physical stress. For example, swimming induces significant trunk muscle atrophy and leads to severe kyphosis, symptoms that resemble those seen in SEPN1-RM patients. Nonetheless, differences in the effects of the *SEPN1* mutation between mice and humans limit the utility of this model for clinical studies [14].

The murine model developed by Moghadaszadeh et al. exhibits a similarly mild phenotype, with normal muscle histology under basal conditions. However, under oxidative stress, such as that induced by vitamin E deficiency, muscle lesions emerge. Moreover, SelN deficiency impairs pulmonary development, resulting in enlarged alveoli, reduced tissue elastance, and increased quasi-static compliance in the lungs of Sepn1^−/−^ mice. These findings suggest a potential pulmonary component to the respiratory complications observed in patients with *SEPN1* mutations [65]. The absence of a spontaneous muscular phenotype in mice may reflect interspecies physiological differences, particularly in muscle usage, as postural muscles are more heavily relied upon in humans than in laboratory mice. Additionally, the environmental conditions of laboratory mice, which limit both activity and stress, may explain the mild pathology observed in SelN-deficient mice. To date, no in vivo observations have linked the physical performance in SelN-deficient mice to changes in SERCA or RyR function [14,62,63,64,65].

### 6.2. Cellular Models

In addition to animal models, cellular systems have provided valuable insights into SelN’s role in maintaining redox balance, calcium homeostasis, and mitochondrial function (Table 1).

One of the earliest studies using HeLa cells with SelN knockdown (KD) via CRISPR/Cas9 revealed that, in the absence of SelN and with overexpression of ERO1, the ER becomes hyperoxidized. This finding suggests that SelN may act as a reductase involved in maintaining ER redox homeostasis. Similarly, Chernorudskiy et al. generated SelN knockout (KO) HeLa cells to investigate SelN’s function as a calcium sensor within the ER and its connection to redox regulation. These KO cells displayed impaired luminal calcium regulation and increased oxidative stress, confirming SelN’s critical role in integrating ER calcium signaling with redox control [8].

Muscle-specific cellular models, such as SelN-deficient murine myoblasts (C_2_C_12_) and primary patient-derived myoblasts, have further elucidated the impact of SelN loss on muscle cell bioenergetics. The absence of SelN was shown to alter ER–mitochondria contact sites (MAMs), leading to impaired oxidative phosphorylation and reduced ATP production. These effects occurred in both muscle (C_2_C_12_ SelN-KO) and non-muscle (HeLa SelN-KO) cell lines, suggesting a conserved role for SelN in mitochondrial–ER communication. Notably, primary myoblasts from SEPN1-RM patients also exhibited MAM disruption and energy deficits, which correlated with the severity of clinical muscle weakness [7]. Further supporting these findings, recently Barraza-Flores used the knockdown (via shRNA) and the knockout (via CRISPR/Cas9) of SelN in C_2_C_12_ cells, reporting significant defects in glutathione homeostasis and mitochondrial metabolism [63]. These results reinforce previous observations and underscore SelN’s essential role in maintaining cellular energy status [7].

In addition to immortalized cell lines, primary cells derived from patients are also used. Primary fibroblasts and myoblasts from young patients harboring *SEPN1* mutations, particularly duplications, single-nucleotide insertions, or other sequence changes causing frameshift, nonsense, initiation codon, or splicing defects [22,49], exhibit alterations predicted to result in premature termination codons, aberrant transcripts, or complete loss of SelN protein expression. These cellular models also displayed increased oxidative and nitrosative stress, elevated ROS and NO production, and extensive protein oxidation, affecting contractile proteins such as actin and myosin heavy chain. Additionally, these patient-derived cells exhibited calcium dysregulation, likely involving redox-sensitive RyR1 channels, and increased susceptibility to oxidative stress-induced cell death [8,49].

## 7. Pharmacological Treatment Perspectives

Targeting the SR stress response represents a promising therapeutic avenue for the treatment of myopathies. However, it is crucial to determine which specific sub-pathways are involved in each disorder. Direct inhibition of SR stress sensors such as IRE1, PERK, and ATF6 may lead to undesirable side effects. A potentially safer approach involves targeting downstream effectors, including eIF2α, GADD34, and ERO1α, or enhancing proper protein folding mechanisms. One example is tauroursodeoxycholic acid (TUDCA), a naturally occurring bile acid synthesized in the liver and approved by the FDA for the treatment of chronic cholestatic liver diseases and gallstones [66]. Clinical studies have shown that treatment with hydrophilic bile acids is generally well tolerated and associated with minimal side effects, indicating a favorable safety profile. Functionally, TUDCA reduces SR and oxidative stress by acting as a chemical chaperone, thereby protecting mitochondria and exerting both anti-apoptotic and cytoprotective effects [49]. In SelN-deficient myotubes, preconditioning with TUDCA has been shown to partially prevent palmitate-induced ER stress and enhance insulin-dependent glucose uptake. Furthermore, chronic TUDCA administration in SelN knockout mice improved diaphragmatic function and restored force production in single muscle fibers, indicating that contractile dysfunction can be fully reversed [40]. Novel ERO1 inhibitors are also effective in SEPN1-RM models [67].

Given the role of SelN in maintaining redox homeostasis, the effects of the antioxidant N-acetylcysteine (NAC) have also been investigated. Studies have reported that NAC can rescue the pathological phenotype in both patient-derived SelN-deficient cells and SelN KO murine models. Based on these findings, a phase II-III pilot clinical trial was launched in 2020, although the results have not yet been published [68,69].

## 8. Conclusions

Studies on mutations in the *SEPN1* gene have become an important topic in biomedical research owing to their extensive influence on skeletal muscle physiology, cellular metabolism, ER stress, and calcium homeostasis. Although animal models have provided valuable insights into the underlying disease mechanisms, they fail to fully recapitulate the complexity of patient phenotypes. This limitation highlights the need for models that more accurately reproduce the pathophysiological features of SEPN1-RM. In this context, patient-derived induced pluripotent stem cells (iPSCs) may represent a promising platform to investigate disease mechanisms and to evaluate therapeutic strategies in a patient-specific manner, as demonstrated for other neuromuscular disorders. In parallel, the development of alternative animal models beyond rodents, including species such as dogs, which are commonly used in myopathy research, could help capture muscle physiology and disease progression that are not fully represented in current models.

Thus, future research should aim to elucidate the molecular mechanisms underlying SEPN1-RM in greater depth while developing models that better reflect the clinical condition to pave the way for the design of targeted therapeutic strategies.

## Figures and Tables

**Table 1:** Summary of animal and cellular models for the assessment of SelN function. The table details the experimental techniques, main phenotypic characteristics and relevant bibliographical references for each model reported. (MAMs = Mitochondria-associated membranes).

Zebrafish		
Techniques applied	Features	References
Antisense morpholino oligonucleotides	Somite disorganization, severe alterations in muscle architecture Defects in slow fiber development Reduced myogenic gene expression in adaxial cells	[58,62]
CRISPR/Cas9	Changes in embryonic muscle function and swimming activity in larvae Co-expression of SelN with genes involved in the glutathione pathway and redox homeostasis	[63]
Murines		
Techniques applied	Features	References
SelN knockout mouse	Mild phenotype with normal muscle histology under basal conditions and increased susceptibility to physical stress Impaired pulmonary development with enlarged alveoli, reduced elastance, and increased lung compliance	[14,64,65]
Hela Cells		
Techniques applied	Features	References
CRISPR/Cas9	Disrupted ER luminal calcium regulation Increased oxidative stress MAM disruption Impaired oxidative phosphorylation Reduced ATP production	[7]
C_2_C_12_ Murine Myoblasts		
Techniques applied	Features	References
CRISPR/Cas9	MAM disruption Impaired oxidative phosphorylation Reduced ATP production Defective glutathione homeostasis Impaired mitochondrial metabolism	[7,63]
Patient-Derived Fibroblasts and Myoblasts		
Techniques applied	Features	References
Patient mutation	MAM disruption Impaired oxidative phosphorylation Reduced ATP production Defective glutathione homeostasis Impaired mitochondrial metabolism	[8,22,49]

## Data Availability

No new data were created or analyzed in this study.

## Abbreviations

The following abbreviations are used in this manuscript:

Sec	Selenocysteine
SEPN1 or SELENON	Selenoprotein N
RSMD1	Rigid spine muscular dystrophy
MmD	Multi-minicore disease
CFTD	Congenital fiber type disproportion
MB-DRMs	Desmin-related myopathy with Mallory body-like inclusions
SEPN1-RM	SEPN1-related myopathy
SECIS	Sec insertion sequence
SRE	Sec codon redefinition element
SBP2	SECIS-binding protein 2
EFSec	Sec elongation factor
ERSE	Endoplasmic reticulum stress response
ER	Endoplasmic reticulum
SR	Sarcoplasmic reticulum
MAMs	Mitochondria-associated membranes
NADH-TR	Nicotinamide Adenine Dinucleotide Tetrazolium Reductase
SDH	Succinate Dehydrogenase
UPR	Unfolded protein response
IRE1	Inositol-requiring enzyme 1
ATF6	Activating transcription factor 6
PERK	Protein kinase RNA-like ER kinase
OXPHOS	Oxidative phosphorylation
TUDCA	Tauroursodeoxycholic acid
NAC	N-acetylcysteine
iPSCs	pluripotent stem cells
BMI	body mass index
